# Psychotherapists’ views on triggering factors for psychological disorders

**DOI:** 10.1007/s44202-022-00058-y

**Published:** 2022-12-12

**Authors:** Eve Riachi, Juha Holma, Aarno Laitila

**Affiliations:** Department of Psychology, University of Jyväskylä, Jyväskylä, Finland

## Abstract

Triggering factors play an important role in the development of psychological disorders. Practicing psychotherapists have valuable knowledge on psychological disorders and since their views on triggering factors have not been reported in the literature, triggers were addressed in this study from psychotherapists’ perspectives. The following three main issues were examined: definitions of triggers, examples of the most recurrent triggers and the idea of a common trigger for psychological disorders. Sixteen psychotherapists agreed to participate in the study. Semi-structured interviews were conducted in person and the data collected were analyzed using frame analysis. Frame analysis aims at representing the data through frames or groups that indicate different interpretations of the same topic. The results showed that the therapists provided three definitions of triggering factors. They most often defined triggers as events, occurrences or situations that explain the onset of psychological symptoms. The psychotherapists also provided examples of triggering factors: these were grouped into three frames, interpersonal, environmental and trauma. The therapists identified no single common trigger, although they discussed common categories and connections between different triggering factors. The findings indicate that triggering factors are complex and closely connected to personal vulnerabilities, as different events and circumstances act as triggers for different individuals. Future research could expand on these findings by examining the constituents of individual vulnerabilities.

## Introduction

Triggering factors were introduced as part of the diathesis-stress model or the vulnerability–stress model in the 1960s [1]. Diathesis or vulnerability referred to psychological and genetic factors that are present in individuals prone to developing a mental disorder. Stress, on the other hand, referred to environmental factors and life events that trigger or aggravate existing predispositions. This model was first utilized to explain the origins of schizophrenia, and was later modified to include psychological disorders in general. In 1980, the term trigger was presented in the third edition of the diagnostic and statistical manual (DSM), specifically in association with post-traumatic stress disorder (PTSD) and its diagnosis [2].

The treatment of psychological disorders is widely based on the origin of disorders. Psychotherapists’ understanding of the causal factors for psychological disorders influences treatment approach [3]. Studies have shown that therapists separate the development of mental disorders into biological and psychological components. Disorders that are viewed to be caused biologically and neurologically form one group, while other disorders seen as caused socially and psychologically make up another [3, 4]. One study showed that psychotherapists perceive mental disorders to range from highly biological to highly psychological or environmental [3]. Another study showed that therapists consider mental disorders with a genetic etiology to have an unfavorable prognosis, and suggested medication as the most effective treatment, in comparison to disorders caused psychologically [5]. The perspectives of psychotherapists regarding the development of disorders has been reported, however, their opinions on triggering factors have not yet been examined. Triggering factors are associated with the development of psychological disorders and require further investigation.

Environmental factors, stressful life events, trauma and neurotransmitter dysregulation are all considered triggers for several disorders [6]. In anxiety disorders, stimuli that lead to intense fear or anxiety, as in the case of phobias or post-traumatic stress disorder, are considered triggers. In turn, the hopelessness theory describes how feelings of hopelessness can trigger depression. A study conducted with a population of adult migrants found that feelings of hopelessness act as a mediator between traumatic childhood experiences and the development of depression [7]. Another study showed that social exclusion triggers paranoia in individuals at high risk for psychosis, concluding that it is how individuals respond to social stressors and not their form or intensity that determines the prevalence of symptoms [8].

Risk factors that increase the probability of developing mental disorders have also been discussed. Examples include genetic vulnerabilities, low socioeconomic status, and family problems [9]. It has been proposed that for a psychological disorder to develop, genetic vulnerability needs an environmental or psychosocial trigger. Several studies have reported risk factors for specific mental disorders. Negative affect and social withdrawal were found to predict the development of eating disorders in adolescents [10]. In the case of depression, such risk factors as sleep problems, abuse, and loss of a partner have been examined [11]. Certain personality traits such as high-ranking neuroticism, negative thinking and low self-esteem have also been reported as risk factors for comorbidity between depression and anxiety [12].

Recent studies have reported on the psychological consequences of COVID-19 and discussed the various symptoms triggered by the pandemic. For example, the occurrence of obsessive–compulsive disorder symptoms, higher stress levels, and generalized anxiety and major depressive disorder symptoms [13, 14]. Other studies have focused on possible triggers of increased stress during the pandemic. For university students, these triggers have been financial problems, social isolation, internet access and changes in educational approaches [15]. The pandemic has also provided evidence on the role of triggering factors in the development of mental disorders. The aim of this study was to gain further understanding of triggering factors that can assist in both the treatment and prevention of psychological symptoms.

While a few studies have addressed triggering factors, less attention has been paid to how triggering factors are defined, and even fewer studies have investigated psychotherapists’ perspectives on triggering factors. Therapists are in daily contact with individuals with mental disorders, and hence their knowledge and experience is invaluable for building knowledge on triggers. A study combining 162 articles on population studies in Europe and America showed that the general public’s attitude towards psychology and psychiatry has improved significantly, and that psychotherapy is chosen over psychiatric medication [16]. The American Psychological Association (APA) reported an increase in the need for mental health practitioners, as well as an increased number of individuals seeking therapy [17]. In addition, two-thirds of psychotherapists included in the study observed an increase in symptom severity among clients in 2022. The increase in demand for psychotherapy emphasizes the need to expand upon the current understanding of mental disorders, including psychological triggers. This study targeted psychotherapists’ views on three main issues: the definition of triggering factors, the most recurrent triggers, and the possibility of a common triggering factor.

## Methods

### Participants and procedures

Sixty Lebanese psychotherapists were contacted either by phone or by email and were asked if they would be willing to take part in this study. Their contact information was obtained from public online information sources used to locate therapists in Lebanon. Lebanese therapists were chosen as participants for this study partly for convenience purposes and partly to decrease cultural biases; since the main researcher collecting the data is Lebanese. Inclusion criteria was working as a psychotherapist, being located in Beirut, and having good English skills. This was especially beneficial, as the interviews were conducted in English. Seventeen therapists responded and agreed to participate in the study, however, one therapist was excluded because they were unable  to meet for the interview. The remaining sixteen psychotherapists, with differing therapeutic and educational backgrounds, constituted the participants for this study. Most had their own psychotherapy clinics, while the others worked in psychotherapy centers or psychiatric units. Table 1 presents the sociodemographic characteristics of participants.

**Table 1:** Sociodemographic characteristics of participants

	Frequency
**Education**	
BA clinical psychology	2
MA clinical psychology	9
Phd clinical psychology	5
**Years of experience as a therapist**	
From 3 to 10 years	7
From 10 to 38 years	9
**Psychotherapy training**	
Cognitive behavioral therapy	7
Integrative therapy	6
Trauma therapy	4
Eclectic therapy	4
Psychodynamic therapy	2
Humanistic therapy	2

Prior to collecting the interview data, participants were informed that their views on the development of psychological disorders would be studied. They were also notified that the interview would be in English and that it would be recorded using a voice recorder. They were also informed that the results of this study would be published. Participants were provided with a copy of the privacy agreement containing a detailed description of the research. After verbally agreeing to these procedures and expressing their willingness to participate, they signed an informed consent form. The written consent form was also signed by the researcher conducting the interviews.

The interview was semi-structured and contained thirteen questions. Five questions were on the participants’ demographics and three on their views regarding triggering factors. The data on the latter three questions were then analyzed. The interview questions are presented in Appendix 1. If needed, additional questions were asked during the interviews for clarification purposes. English was chosen as the interview language both to decrease translation bias and to utilize participants’ choice of words and phrases in their perception of triggering factors.

The interviews were conducted in person and each participant was interviewed separately. The interviews took around 25 min on average and were recorded using a voice recorder and then transcribed. One interview was collected directly in writing as the participant preferred not to have their voice recorded. Apart from one interview that was mostly conducted in Arabic and subsequently translated into English, all the interview data were conducted in English. Some therapists also occasionally used Arabic to provide additional explanations and examples; these were also translated into English. Participants’ personal information was protected throughout the study and their names were not included in the saved data. Participants were aware that their participation was voluntary and received a copy of the signed consent form.

### Analysis

A few qualitative research methodologies were discussed in regards to the analysis of this study, such as grounded theory, content analysis and frame analysis. Frame analysis [18] was used to investigate psychotherapists’ views on factors triggering psychological disorders. Grounded theory was ruled out since the aim of the study focused more on understanding therapists’ perspectives and less on creating a theory on triggers [19]. As for content analysis, it was also not fitting for the scope of this study because therapist’s opinions were not interpreted for the purpose of extracting underlying meaning. However, through frame analysis, participants’ opinions were grouped to identify different interpretations of triggering factors.

Frame analysis was chosen because it allows examination of how certain issues are understood differently based on perception. Within frame analysis, the concept of understanding emerges from grouping social situations based on an individual frame of reference [18]. Therapists’ opinions for examples could be shaped by their therapeutic approach, psychotherapy training, clinical experience and even personal knowledge [20]. Hence, utilizing frame analysis allowed the exploration of diverse outlooks therapists have regarding triggering factors.

Three interview questions were analyzed using Atlas.ti software. The data for each interview question was coded separately. The codes were created by extracting phrases and terminology used by the psychotherapists when discussing triggers. Similar explanations or codes were then grouped together to form frames. Two interviews were excluded from the analysis of the second research question as these participants’ answers did not provide specific information on or examples of recurrent triggers.

The first author was responsible for conducting the interviews, for data collection, and for initial coding. The codes were then analyzed and interpreted in collaboration with the other two authors. The cooperation between authors occurred during regular meetings which included a detailed discussion regarding coding, agreement on the most representative titles for frames and appropriate placement of the codes within each frame. The first author holds a master’s degree in clinical psychology with supervised psychotherapy training in several therapeutic approaches with no specific focus. The other authors had received psychotherapeutic training in systemic therapy, utilize an integrative approach in their clinical practice and have substantial experience in qualitative research.

## Results

### Definitions of triggering factors

The first research question focused on the definition of triggers or triggering factors. The psychotherapists were asked to define what they considered constituted a triggering factor. Analysis of the data yielded three definitions or frames that were labeled situational, past experiences, and non-specific. Table 2 presents the three frames and the number of times each definition was mentioned by the psychotherapists. The most frequently mentioned was the situational frame.

**Table 2:** Frequencies of the frames describing the definition of triggers

Definition of triggers	Frequency
Situational frame	8
Past experiences frame	4
Non-specific frame	4
Totals	16

### Situational frame

The triggers that were mentioned in this frame included, in the words used by the interviewee, events, incidents, changes, new situations, circumstances, happenings in the environment, and something individuals are exposed to. The occurrence of events could be either in the past or the present. Past changes or events occurring at the time of emergence of psychological symptoms were for instance divorce experienced in childhood, whereas present events were for instance relocating to a new country.

The effects of triggers as described by the therapists were activation of psychological symptoms leading to distress on an emotional, psychological or physical level, resulting in dysfunction or feelings of loss of sense of control as well as stimulating a system of thinking and behaving. Moreover, the therapists explained that the effects of a triggering factor are dependent on the individual’s vulnerabilities and capacity for resilience.

- *Interview 3: I try to identify the moment where the symptoms started appearing and when we have these moments or this period of life, we try to identify what has changed, what has happened at that point in time; this would be the triggering factor for the symptoms.*
- *Interview 5: Triggers come from the environment; from a new situation; something you couldn’t deal with.*
- *Interview 7: A triggering factor would be a change; any sort of change can trigger a psychological disorder.*

### Past experiences frame

The psychotherapists identified another definition for triggering factors as elements of the present environment that resembled negative events experienced by the individual in the past. Some therapists used expressions such as the present environment or present happenings. Past experiences were described by phrases such as negative past experiences, unfulfilled emotional needs from childhood, and past trauma. Some therapists stated that triggers could be minor events that nevertheless generate a huge reaction in individuals. This reaction, linked to past experiences, is thus not usually proportional to the present triggering event. Examples of triggers that reminded individuals of past trauma included songs, smells, or another person’s personality or tone of voice. Other examples were situations resembling past negative experiences such as not feeling listened to or being ignored. Yet other examples focused on childhood experiences and revolved around issues of abandonment, or inadequacy.

The association between triggers and the brain or the central nervous system was also discussed. The effects of triggering factors were described as arousing the central nervous system, leading to a psychological, emotional or behavioral reaction as well as raising tension and possibly leading to psychological symptoms.

- *Interview 11: An element in your environment, in the present, that is somehow similar either in setting, in content, or in the personality of the individuals around you to a previous actual trauma that you have gone through.*
- *Interview 14: A triggering factor is anything that might happen in the present that stimulates something in the person’s mind or feelings or even behavior. For example, when something happens in the present and it just reminds the person of a negative experience in the past, the brain links that together and the experience in the past with all its cognitions and feelings are relived in the present…the person might feel very upset and this wouldn’t usually be proportional to what is happening in the present.*

### Non-specific frame

The triggers in this frame remained undefined or vague. The therapists provided a general definition of a trigger and focused on its effects rather than labeling it or specifying the elements a triggering factor. Therapists used words such as a factor, anything or something in their definitions. They described a trigger as something normal that has a strong effect on particular individuals. Such effects included inducing a psychological disorder, activating symptoms, and causing dysfunction or loss of control.

- *Interview 1: Factors that activate symptoms, contribute to its beginning, or activates the disorder in some way.*
- *Interview 9: Something that makes the person go out of control, when others around might see it as something very normal, but it makes the person completely dysfunctional.*

### Most recurrent triggering factors

The second research question aimed at eliciting the most recurrent triggering factors. The psychotherapists were asked to describe and exemplify the triggers they had encountered most often in their clinical practice. All mentions of these triggers were extracted from the data and grouped into three frames: interpersonal, environmental, and trauma. Table 3 presents the three frames and their frequency of occurrence in the data. The interpersonal frame contained the most mentioned triggers.

**Table 3:** Frequencies of the frames comprising the most recurrent triggering factors

Most recurrent triggers	Frequency
Interpersonal frame	13
Environmental frame	9
Trauma frame	7
Totals	29

### Interpersonal frame

The most recurrent interpersonal triggering factors mentioned by the interviewees included relationship issues such as break-ups, being in a difficult relationship, losing trust in a relationship, and the death of a loved one. The therapists also mentioned unsupportive family or societal support systems as well as dysfunctional support systems. Personality differences, whether between parents and children or between couples, that create problematic interactions were also mentioned. This frame also included family issues and unmet childhood needs, such as children not feeling understood by their families or not receiving enough attention, care and emotional support from their parents. Unmet childhood needs were discussed in relation to negative cognitions and individuals’ perceptions of themselves and the world.

- *Interview 6: You know triggering factors are numerous, if I want to put them in categories or if I want to summarize what I have observed, it is most of the time, if not always, related to interpersonal issues. Loss for example, let’s say losing someone or breaking ups, having difficult relationships with people, losing trust. So in most of the cases, if not in all the cases, it is about relationships and interpersonal issues.*
- *Interview 7: In my experience, triggers are relational in nature and are based on repetitive ways of being related to during times of need. For example, most people come to therapy and when we explore their past, we would see common patterns of either neglect or an inability of their caregiver to show a deeper understanding or an inability to cope with the child's emotions or basic needs at the time…Being too rigid or flexible with the child or having unrealistic expectations from the child, not giving them enough advice …that creates a level of frustration and results in core beliefs such as: I am not lovable, I am not worthy, or I don’t have what it takes to make it in life.*
- *Interview 15: A lot of it is relational, so people. People trigger other people.*

### Environmental frame

The main examples in this frame were challenging situations, stressful life events, or changes that an individual is subjected to. Examples of environmental triggers given by the therapists included financial difficulties, unemployment, poverty, accidents, being the victim of a crime such as robbery, changes in an individual’s environment such as relocating to a new country, changing schools or jobs, and developmental or health-related changes like an illness. The absence of certain coping skills or having poor coping skills in certain areas of life were offered as possible reasons why certain events are challenging and become triggers for some people and not others. This frame represents factors that are not based on human interaction, which is noticeably distinct from the interpersonal frame. Many environmental triggers were included except for trauma related triggers which constituted the third frame in this study.

- *Interview 2: Changing of the environment, like relocating to different places, difficulty integrating usually is a big problem, like changing schools, changing jobs, changing countries.*
- *Interview 14: Typically, the most recurrent triggers would be a challenging situation for this specific person who perhaps has poor coping skills in that specific area.*
- *Interview 15: Poverty in this situation, specifically within the population that I work with. Their constant inability to make ends meet, not having a job, trying to find a job; it triggers a lot of things such as anxiety and depression.*

### Trauma frame

Triggering factors related to trauma or abuse were considered by the therapists in this frame to be the most recurrent triggers they had encountered in their clinics. They cited such factors as emotional, verbal, or sexual abuse, neglect, traumatic events such as war or torture, and childhood traumas resulting from bullying or witnessing intense conflicts between parents. The therapists also stated that anything that reminds the individual of a previously experienced trauma could act as a trigger. Such reminders included hearing a certain tone of voice, watching a movie, being in a new job in a new setting or the personality of a boss or close individual.

- *Interview 8: Childhood traumas, traumatic events like bullying, sexual or physical abuse, intense conflicts between parents, for example if the child is seeing their father hitting their mother.*
- *Interview 12: Abuse, because I have several clients dealing with the issue of emotional, verbal, or sexual abuse or even neglect and some have more than one experience of abuse.*
- *Interview 13: The trigger could be anything. There is no way to specify, it depends on the person’s actual trauma, some people are triggered by the personality of their boss or their husband. Some people are triggered by the tone of voice, or the color of someone’s hair. Sometimes a similar setting can be a trigger; for example, going to a new office and working in a new setting can be a trigger for previous separation anxiety or abandonment trauma in kindergarten or preschool. Sometimes when you are dealing with someone who is authoritarian like a boss, and one of your parents was also authoritarian, then the boss will be a trigger. Also movies can be triggers, it doesn’t have to be a person or a setting. It could be a movie.*

### Common trigger

To answer the third research question, the psychotherapists were asked whether or not they thought there was a common triggering factor for most psychological disorders. They gave different answers and showed different ways of understanding the question. Similar answers were grouped together, yielding three frames: connectivity, categorization and dismissive. Within all three frames therapists seemed to disagree with the idea of a single common trigger. Table 4 presents the three frames and their frequencies. The connectivity frame was the most frequent.

**Table 4:** Frequencies of the frames in response to the question about a common triggering factor

Is there a common trigger?	Frequency
1. Connectivity frame	9
Common outcome	7
Common cause	2
2. Categorization frame	6
3. Dismissive frame	2
Totals	17

### Connectivity frame

In this frame, the psychotherapists focused on the common features of triggers and gave explanations of what, in their opinion, connects triggers together. Their understanding of the question regarding the presence of a common trigger focused on commonality, such as a common cause for triggers or a common reaction that triggers provoke in individuals. Even though some therapists clearly stated that they do not think there is one common trigger, they still provided common aspects for triggers. Two subcategories were mentioned explaining the connections between triggers: common outcome and common cause. Therapists mentioned the following common outcomes of triggers: distress, feelings of vulnerability, helplessness and confusion, losing control and losing one’s identity. Another connector they mentioned was a common cause or a common origin for most triggering factors which is past unresolved trauma.

- *Interview 3: What is common with triggers is confusion and a sense of not knowing what to do and not having control…it is when people want to change a specific situation but they can’t so that gets confusing and they start having problems.*
- *Interview 6: There is a common cause for triggers which is trauma, but no common trigger.*
- *Interview 7: I think every individual is different, so every trigger is different, but the common denominator would be that it causes distress. Whatever the situation is and whatever the trigger may be, it all leads to the person feeling distress.*
- *Interview 12: Definitely there has to be something in common, it is the point where something happens to you and you get lost. What triggers have  in common is a situation which makes me vulnerable, regardless of the type of trigger, a situation that puts me in a certain state of mind where I feel maybe helpless, I feel that I am not in control anymore. That is the trigger, something that pushed me and I cannot. It is the reaction that people have in common, disregarding the differences, it is the fact that I had enough.*

### Categorization frame

In this frame, common themes or common categories were mentioned in response to the question of whether psychological disorders have a common trigger. Psychotherapists explained that while there is no single specific common triggering factor, triggers have common elements that they have observed in their clinics. Thus, although the views in this frame discounted the idea of one common trigger, they nevertheless emphasized that triggers shared common themes, such as the family, the environment, traumatic events, and unmet needs. Other common categories were interpersonal issues, recurrent negative experiences, abuse, as well as issues related to power, safety or security, love and acceptance, self-competence or self-worth, and responsibility.

- *Interview 2: I do believe that what is common in most triggers of psychological disorders is unmet needs but I cannot discount biology, so for that reason I couldn’t say there is a common trigger. For me the answer would be no, but I would say that a well-adjusted family is certainly a protective factor.*
- *Interview 4: Not a common trigger, but there are common themes. So it is not one common trigger but there are common themes which are related to self-competence, love and acceptance, safety and danger.*
- *Interview 14: It is interpersonal issues and interpersonal problems. You could also be dealing with something else, for example you may have problems with achievement or with self-esteem but then again self-esteem is basically how you were looked at when you were a child, whether you were praised or criticized. It goes back to interpersonal issues.*
- *Interview 16: Traumatic events, but not for all, not in general. I believe there isn’t one common triggering factor, several factors play a role. One of the major ones is traumatic events amongst others.*

### Dismissive frame

Therapists in this frame dismissed the idea of a common triggering factor. They did not provide further explanation other than it would be too reductionist to suggest a single common trigger for psychological disorders.

## Discussion

The aim of this study was to identify the viewpoints of psychotherapists on triggering factors. Three main issues were addressed: the definition of a trigger, examples of the most recurrent triggers, and the existence of a common trigger. First, different definitions for a triggering factor were grouped into three frames: situational, past experiences and non-specific. In the situational frame, which was the most frequently mentioned, triggering factors were seen as circumstances or events that individuals are exposed to. In another definition, triggers were seen as elements in the present environment that resemble past negative experiences. Second, the psychotherapists’ views on the most recurrent triggering factors were grouped into three frames: interpersonal, environmental and trauma. The interpersonal frame was the most frequently mentioned and included triggers relating to relationships with others, family issues, personality differences and dysfunctional support systems. Examples of triggers relating to environmental circumstances or trauma constituted the other two frames. The third research question focused on whether or not psychotherapists considered that mental disorders were triggered by a common factor. The answers seemed to differ, according to the way the question was understood, and were grouped into three frames: connectivity, categorization and dismissive. The connectivity frame included two subgroups for connections the therapists considered to be common for most triggers, which were common outcome and common cause.

What constitutes a triggering factor has not been precisely defined in previous studies. The terms trigger and risk factor seemed to be used interchangeably. According to previous research, while risk factors do not necessarily induce symptoms, and certain risk factors such as genetics need a trigger in order for a disorder to develop, the accumulation of risk factors could lead to the development of symptoms [9]. In this study, the definitions the psychotherapists offered for triggering factors yielded a different classification from that of risk factors. First, the psychotherapists argued that the triggers occurred immediately prior to the onset of symptoms. Moreover, the majority of the therapists seemed to agree on the effects of triggering factors, which included distress, dysfunction, and psychological symptoms, resulting in an emotional, psychological, or physical reaction, and loss of sense of control. Some therapists in this study also discussed the accumulation of negative events or the buildup of unprocessed traumas which could become a trigger. They explained that a triggering factor is not necessarily a huge event; it may sometimes be the addition of a minor event that pushes the individual over the edge.

Although the therapists in this study defined triggering factors in a way that distinguishes them from risk factors, the examples they gave showed some similarities. The examples of triggers and risk factors given in the literature include environmental factors, stressful life events, social stress, social withdrawal, trauma. abuse, family issues, low socioeconomic status, genetic vulnerabilities, neurotransmitter imbalance, feelings of hopelessness, negative affect, personality traits, sleep problems, and loss of a partner [6–12]. The psychotherapists in this study also mentioned family issues, poverty, environmental changes, stressful life events, death of a loved one, trauma, abuse, feelings of helplessness and personality differences as triggering factors. However, they did not mention genetic vulnerabilities or neurotransmitter imbalance as triggering factors. The triggers they focused on all fell within the interpersonal, environmental and trauma frames, while they made no mention of neuropsychological or medical factors, which would mainly be classified as risk factors.

Earlier research showed that social stress can trigger paranoia in individuals at risk for psychotic disorders, explaining that it is the individual’s reaction to a stressor that determines the prevalence of the symptoms and not the type or intensity of the stressor [8]. The psychotherapists in this study complemented this perspective by arguing that triggers could be normal events that, however, affect specific individuals differently, precisely because of their individual vulnerabilities. For some therapists these included weak coping skills in certain areas of life such as work, personal relationships, or family. Others focused on past negative experiences and their role in shaping an individual’s cognitions, perceptions and emotional reactions. Some also mentioned past unresolved trauma as the origin of most triggers.

A recent study of feelings of hopelessness in an adult migrant population found that hopelessness acted as a mediator between childhood traumatic events and depressive symptoms [7]. The hopelessness theory similarly explains how hopelessness can affect the development of depressive symptoms [6]. In this study, the psychotherapists stated that most triggers have connections with feelings such as distress, vulnerability, helplessness and confusion, loss of sense of control, and loss of identity. Moreover, most triggers were also connected with past unresolved trauma. Although the psychotherapists did not use the word hopelessness, their opinions were clearly informed by a similar notion.

In this study, therapists defined triggering factors as events, situations or occurrences which are linked to past experiences and that activate intense reactions, psychological symptoms or even disorders. This definition combines aspects from the three frames provided by the therapists. In addition, some therapists discussed triggers in relation to psychotherapy and stated that the client’s explanation of their problem is generally different from the reason they are suffering or having difficulties. During their first session, clients are asked why they have come to therapy and they answer providing an explanation of the issues they are facing. However, the factors triggering their problems are not always clear to either the client or the therapist. Throughout therapy, triggers reveal themselves. This process is dependent on the client’s willingness to discuss their life surrounding the onset of their symptoms, including events that they might experience as uncomfortable and challenging. During therapy sessions, something said or done by the therapist may also trigger reactions in clients. Such occurrences are also important elements of therapy, as they create an opportunity for both the therapist and the client to discuss the situation, understand what it was that had bothered the client, learn about the client’s triggers, and treating past experiences linked to the trigger. An important part of resolving triggers is achieved through emotionally processing past experiences in a healthy way; which renders past traumatic experiences into just memories [21].

With respect to recurrent triggers, those that were most often mentioned were found in the interpersonal frame. Abuse and neglect were viewed by some therapists as traumas per se, while others categorized neglect as part of unmet childhood needs, which is also a trigger in the interpersonal frame. Other triggers related to past traumas, such as the personality of others or being criticized by someone, might also be considered as interpersonal. Some therapists explained that some issues, such as experiencing performance anxiety at work, are connected to self-esteem which, in turn, is generally based on how much an individual was criticized or praised when growing up. Even an environmental factor such as moving to another country, could also be considered as interpersonal trigger, especially if it is associated with difficulties in adjusting or relating to a new social setting. These findings indicate that several triggers might originate in relationships with others, thus testifying to the importance of past and present interpersonal interactions.

The environmental and trauma frames were also seen as important. For example, war and poverty were discussed as examples of triggers in both the environmental and trauma frames. This overlapping between frames demonstrates the difficulty of categorizing life events and individual experiences, which act as triggers for different reasons across individuals, and further indicates the link between triggers and individual vulnerability.

The psychotherapists seemed to understand the third research question differently. They were asked whether or not they thought psychological disorders share a common trigger. Some stated that triggers have common outcomes or causes, others that common categories of triggers exist, such as the family, the environment or trauma, and many mentioned that there is no single common trigger. The uncertainty in the therapists’ answers demonstrates their striving to maintain an integrative approach as well as their unwillingness to limit attributing the onset of psychological disorders to a single common trigger.

The therapists suggested common themes and connections between triggers which also indicated their complexity. Common categories included interpersonal issues, unmet needs, recurrent negative experiences, abuse, and issues related to power, safety or security, love and acceptance, self-competence or self-worth, and responsibility. Others stated that triggers fall under the main headings of family, trauma, and the environment. On the issue of common outcomes of triggers, the therapists stated that triggers lead to distress, feelings of vulnerability, helplessness and confusion, loss of control and loss of one’s identity. Another common feature underlying triggers was past unresolved trauma. These findings show that while triggers have common effects on individuals and could belong to similar categories, each case needs to be assessed individually.

The COVID-19 pandemic could be considered an environmental trigger since it originated from situational events. It is likely that the pandemic will have a lingering and prolonged influence on the occurrence of psychological symptoms and disorders. Several of the shared outcomes of triggers, such as feelings of distress, vulnerability, helplessness, confusion, loss of control, and loss of one’s identity, discussed in this study were present during the pandemic. The unpredictability and apparent uncontrollability of the pandemic might have also induced a sense of hopelessness. The restoration of psychological health occurs, as a combined effort between therapists and their clients, in creating an opportunity for clients to reconnect with their sense of control and mastery. Whether through learning new coping skills or better understanding and perceiving their own power, individuals need to be able to visualize their choices instead of being on the receiving end and simply reacting to what is happening. This, in turn, facilitates people’s capacity to adjust and cope, even in the face of unpredictable and uncontrollable triggers.

## Strengths and limitations

Previous research concerning psychotherapists’ viewpoints on triggering factors is limited. This study provided novel and abundant information on this topic. The participating psychotherapists communicated their thoughts and perspectives on triggering factors, offering definitions of triggers along with examples and themes. Connections between triggers were also discussed in addition to the relation between triggering factors and individual vulnerability.

This study also created an opportunity to understand triggering factors from a comprehensive point of view, especially that the participation of psychotherapists was voluntary, and analysis of the data was done irrespective of the participants’ therapeutic approaches.

This study has its limitations. First, the frames in which the different examples of triggers were collected seemed to overlap. The most recurrent triggers were distributed in three frames ‒ interpersonal, environmental and trauma ‒ although some could similarly be in more than one frame. Second, two interviews were omitted from the data, as they lacked concrete examples.

Although English is not the native language of the participants in this study, the use of English in the interviews is not considered a limitation. Most of the participants had either studied or worked in English and some had received training and also taught in English at university level. Only one interview was translated from Arabic into English, and translations were otherwise minimal. However, the differences in the therapists’ understanding of the third research question may be linked to their English language skills. Although, it is also possible that therapists’ answers differed not because they had misunderstood but because their perspectives and their way of interpreting the question were different. Moreover, their different responses yielded valuable knowledge on triggering factors and enriched the findings of this study.

## Conclusion

This study examined the viewpoints of psychotherapists on triggering factors. The data yielded information on the definitions of a triggering factor, the most recurrent triggers, and therapists’ views on the existence of a common trigger. The results showed that triggering factors were considered as events, situations or occurrences that are linked to past experiences and that explain the onset of individuals’ problems or symptoms. Triggering factors are often everyday events that are triggers for some people but not others, as triggers are closely connected to personal vulnerabilities such as poor coping skills, negative experiences, or past trauma. Finally, while the therapists identified no single common trigger, they discussed common categories and links between triggering factors.

Future research could expand on the findings of this study and examine the factors underlying triggers. Understanding the constituents of individual vulnerability would provide valuable information. Recognizing the reasons why certain kinds of events are triggering could aid in both prevention and treatment. Moreover, with respect to the feelings of helplessness, vulnerability and loss of control that triggers evoke, it would be interesting to examine the ways in which therapists could help their clients regain their sense of control and mastery.

## Data Availability

The datasets analyzed during the current study are not publicly available due to the absence of consent from participants for absolute distribution of the data.
